# Phytochemical Properties and Anti-Proliferative Activity of *Olea europaea* L*.* Leaf Extracts against Pancreatic Cancer Cells

**DOI:** 10.3390/molecules200712992

**Published:** 2015-07-17

**Authors:** Chloe D. Goldsmith, Quan V. Vuong, Elham Sadeqzadeh, Costas E. Stathopoulos, Paul D. Roach, Christopher J. Scarlett

**Affiliations:** 1 Nutrition Food & Health Research Group, School of Environmental and Life Sciences, University of Newcastle, Ourimbah, NSW 2258, Australia; E-Mails: vanquan.vuong@newcastle.edu.au (Q.V.V.); paul.roach@newcastle.edu.au (P.D.R.); c.scarlett@newcastle.edu.au (C.J.S.); 2 School of Biomedical Sciences and Pharmacy, University of Newcastle, Ourimbah, NSW 2258, Australia; E-Mail: elham.sadeqzadeh@newcastle.edu.au; 3 Faculty of Bioscience Engineering, Ghent University Global Campus, Incheon 406-840, Korea; E-Mail: costas.stathopoulos@ghent.ac.kr

**Keywords:** olive leaf, oleuropein, pancreatic cancer, phenolic compounds, *Olea europaea* L., antioxidant activity, phytochemicals, biophenols

## Abstract

*Olea europaea* L*.* leaves are an agricultural waste product with a high concentration of phenolic compounds; especially oleuropein. Oleuropein has been shown to exhibit anti-proliferative activity against a number of cancer types. However, they have not been tested against pancreatic cancer, the fifth leading cause of cancer related death in Western countries. Therefore, water, 50% ethanol and 50% methanol extracts of *Corregiola* and *Frantoio* variety *Olea europaea* L*.* leaves were investigated for their total phenolic compounds, total flavonoids and oleuropein content, antioxidant capacity and anti-proliferative activity against MiaPaCa-2 pancreatic cancer cells. The extracts only had slight differences in their phytochemical properties, and at 100 and 200 μg/mL, all decreased the viability of the pancreatic cancer cells relative to controls. At 50 μg/mL, the water extract from the *Corregiola* leaves exhibited the highest anti-proliferative activity with the effect possibly due to early eluting HPLC peaks. For this reason, olive leaf extracts warrant further investigation into their potential anti-pancreatic cancer benefits.

## 1. Introduction

*Olea europaea* L*.* leaf (olive leaf) is a waste product of the olive oil extraction process, weighing up to 10% of the material arriving at the mill. Currently, this by-product is not profitable; olive leaves are often used as animal feed or simply burned with excess branches gathered from pruning [1,2]. Many olive oil producers even charge a fee to the olive farmer for the disposal of olive leaves. The interest in olive leaf has grown in recent years due to the high concentration of phenolic compounds, of which oleuropein is the most abundant. A number of the health benefits of virgin olive oil consumption have been attributed to oleuropein. It has been found to have anti-atherogenic [3], anti-inflammatory [4] and antimicrobial [5] properties. More recently, oleuropein has been investigated for its potent anti-cancer activity. It has been shown to inhibit proliferation and migration of a number of advanced grade human tumour cell lines in a dose dependent manner [6,7,8,9,10,11]. However, the effect of olive phenolic compounds has yet to be investigated for pancreatic cancer.

Pancreatic cancer is a devastating heterogeneous disease with significant resistance to the limited conventional treatment options and the current chemotherapy agents are highly toxic [12,13,14,15]. Thus, it is essential to control and manage the development of pancreatic cancer [16] as well as to develop novel therapeutic strategies against it. The use of olive phenolic compounds may serve as a useful strategy to inhibit carcinogenesis [14]. To our knowledge there has not been any investigation into the effect of olive leaf phenolic compounds on pancreatic cancer cells.

It is important to understand the effect different extraction conditions have on phenolic compound yield. A number of methods have been proposed for the extraction of phenolic compounds from olive leaves [17,18,19]. However, it is difficult to compare these studies since they use very different methods including advanced technologies and an array of different solvents. Furthermore, the use of advanced technologies, including microwave and ultrasound-assisted extraction methods, are difficult to scale up to an industrial setting and organic solvents can be expensive and difficult to dispose of. This has led to a push from industry and researchers for the development of more environmentally friendly, or “green” extraction techniques, for example, using water as an extraction solvent. However, it is important to understand the efficacy of these “green” extraction protocols compared to organic solvent extraction methods and advanced technologies.

We hypothesised that water is an effective extraction solvent for preparing oleuropein rich olive leaf extracts with anti-pancreatic cancer activity. Therefore, this study aimed to characterise the phytochemical properties of olive leaf extracts obtained from two different cultivars of olive leaves via different previously optimised extraction methods. A water extraction method was compared to two ultrasound-assisted extraction methods with 50% ethanol or 50% methanol as the solvent. The anti-pancreatic cancer effect of these extracts was also assessed.

## 2. Results and Discussion

There were no differences between the Corriola and Frantoio varieties in their TPC, total flavonoids and oleuropein content (Table 1) and in their antioxidant capacity (Table 2). Many variables can affect the phenolic compound content of olive products including the position on the tree, cultivar, soil mineral content as well as sun exposure. However, the geographic location of the tree has been shown to have the largest effect on the phenolic compound profile of olive products [20]. Therefore, it is likely that the reason that no difference was seen between the two different varieties of olive leaves was that they were from the same location.

**Table 1:** Phytochemical properties of olive leaf extracts. Total phenolic compounds (TPC) are expressed as gallic acid equivalents (GAE)/g of extract, total flavonoids are expressed as rutin equivalents (RE)/g of extract and oleuropein is expressed as mmol/g of dried extract.

Solvent	Cultivar	TPC	Total Flavonoids	Oleuropein
		(mg GAE/g)	(mg RE/g)	(µmol/g)
Water	*Corregiola*	230.15 ± 6.85 ^a^	345.45 ± 85.71 ^a^	86.33 ± 1.41 ^a^
Ethanol (50%)	*Corregiola*	238.70 ± 11.85 ^a^	828.13 ± 47.82 ^b^	114.54 ± 1.14 ^b^
Methanol (50%)	*Corregiola*	231.05 ± 11.15 ^a^	539.53 ± 18.16 ^a^	109.54 ± 3.92 ^b^
Water	*Frantoio*	233.45 ± 0.20 ^a^	442.95 ± 16.52 ^a^	85.11 ± 1.65 ^a^
Ethanol (50%)	*Frantoio*	241.60 ± 23.5 ^a^	1035.79 ± 121.25 ^b^	111.93 ± 5.80 ^b^
Methanol (50%)	*Frantoio*	236.20 ± 11.02 ^a^	528.51 ± 43.87 ^a^	105.01 ± 1.13 ^b^

^a,b^ Values in the same column not having the same superscript letter are significantly different from each other (*p* < 0.05).

**Table 2:** Antioxidant capacity of olive leaf extracts measured using three different antioxidant activity assays. DPPH is expressed as % inhibition and FRAP and CUPRAC are expressed as mg trolox equivalents (TRE)/g of dried extract.

Solvent	Cultivar	DPPH	FRAP	CUPRAC
		(% Inhibition)	(mg TRE/g)	(mg TRE/g)
Water	*Corregiola*	74.75 ± 5.85 ^a^	22.85 ± 19.17 ^a^	308.65 ± 36.83 ^a^
Ethanol (50%)	*Corregiola*	70.97 ± 12.9 ^a^	218.51 ± 49.34 ^a^	322.32 ± 32.99 ^a^
Methanol (50%)	*Corregiola*	84.25 ± 4.31 ^a^	237.81 ± 35.49 ^a^	302.54 ± 6.75 ^a^
Water	*Frantoio*	75.61 ± 2.73 ^a^	232.12 ± 4.89 ^a^	318.07 ± 59.76 ^a^
Ethanol (50%)	*Frantoio*	86.34 ± 4.27 ^a^	303.44 ± 19.81 ^a^	326.62 ± 21.71 ^a^
Methanol (50%)	*Frantoio*	86.63 ± 8.19 ^a^	216.15 ± 55.66 ^a^	303.92 ± 22.17 ^a^

^a^ All values in the same column were not significantly different from each other (*p* > 0.05).

### 2.1. The Influence of Extraction Methods on Phytochemical Properties

The different solvents and extraction conditions had no effect on the TPC but did influence the total flavonoids and the oleuropein content in the extracts (Table 1). The water extract had a lower level of total flavonoids and oleuropein while the methanol extract had a lower level of total flavonoids, when compared to the ethanol extract. Flavonoids are the largest group of phenolic compounds and include both polar and non-polar moieties. The 50% ethanol extract contained more than double the total flavonoids compared to the water extract (Table 1). This suggests that the majority of the compounds present in the olive leaves were either less polar flavonoids or were potentially heat sensitive compounds which were degraded during the water extraction process conducted at 90 °C for 70 min [21]. Consistent with this, acetone is well known to be the best solvent for the extraction of flavonoids [21] and acetone is less polar than 50% ethanol.

**Figure 1:**
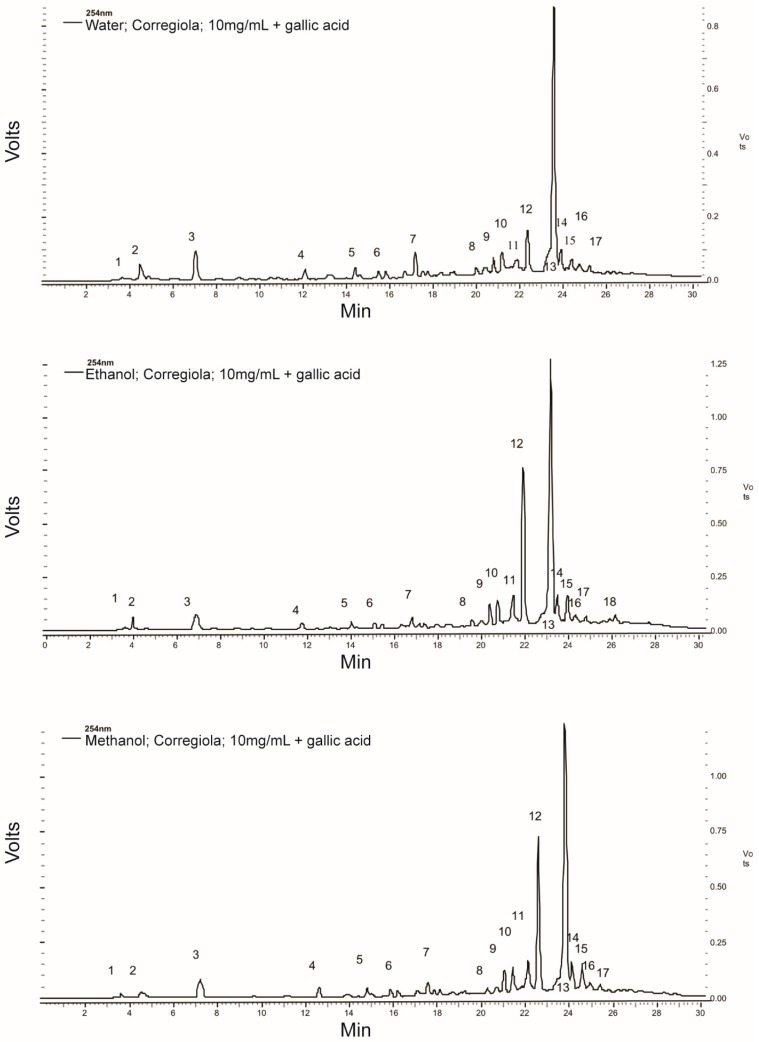
Typical HPLC chromatograms for the water, 50% ethanol and 50% methanol extracts from olive leaves. Peaks identified were: (3) gallic acid (internal standard), (5) tyrosol and (13) oleuropein.

The typical HPLC chromatograms for the different extraction protocols (Figure 1) showed similar peak profiles. However, the earlier-eluting less polar peaks (1–7) were slightly larger in the water extract while the later-eluting more polar peaks (9–17) were larger in the ethanol and methanol extracts. The greatest effect was seen in peak 12, for which the area was more than tripled in the ethanol and methanol extracts compared to the water extract.

Since oleuropein (peak 13) is one of the less polar compounds in the HPLC chromatograms (Figure 1), it was not surprising to see that the organic solvents extracted more of it from the olive leaves. However, the water method still extracted approximately 80% of the oleuropein compared to the ethanol extraction method. Moreover, there was no difference in the level of TPC between the different extracts. Therefore, when considering the current push towards “green” extraction protocols, the water extraction method [21] is an excellent candidate for industrialisation.

### 2.2. The Influence of Extraction Methods on Antioxidant Capacity

Despite the differences in the phytochemical properties of the extracts obtained with the different solvents, there was no significant difference in their antioxidant capacity as measured via the DPPH, FRAP or CUPRAC assays (Table 2). This was not surprising since although individual compounds did vary depending on the extraction conditions, there was no difference in the TPC of the different extracts (Table 1). This further highlights the effectiveness of this “green” water extraction protocol. Additionally, previous reports have shown that antioxidant activity can increase in extracts whose high molecular weight compounds have degraded into more active lower molecular weight compounds. One example of this is the degradation of oleuropein into hydroxytyrosol and tyrosol. However, this seems unlikely since tyrosol (peak 5) was not detected in significant amounts and hydroxytyrosol was not detected at all in the HPLC chromatograms (Figure 1).

### 2.3. The Influence of Extraction Methods on Growth Inhibition of Pancreatic Cancer Cells in Vitro

At a concentration of 200 µg/mL, all of the crude olive leaf extracts for both the *Corregiola* and *Frantoio* cultivars were able to reduce the viability of the MiaPaCa-2 cells to less than 1% relative to controls, and they were significantly more toxic (47.8%) than the chemotherapy drug gemcitabine at its IC_50_ of 50 nm (Table 3). At 100 µg/mL, the water extract of the *Frantoio* variety (0.47%), the 50% ethanol extract of the *Corregiola* variety (4.26%) and the water extract of the *Corregiola* variety (14.59%) demonstrated a significantly greater effect on the cell viability of the MiaPaCa-2 cells compared to the 50% ethanol (30.37%) and the 50% methanol (41.13%) extracts of the *Frantoio* variety and the 50% methanol extract of the *Corregiola* variety (32.83%). However, all the extracts at 100 µg/mL were still more toxic than gemcitabine at its IC_50_ (Table 3).

**Table 3:** Anti-proliferative activity of olive leaf extracts (0–200 µg/mL) on MiaPaCa-2 pancreatic cancer cells. Results are expressed as % viability compared to controls ± standard deviation.

Solvent	Cultivar	Gemcitabine (50 nM)	Concentration of Olive Leaf Extract (µg/mL)			
			0 (Controls)	50	100	200
water	*Corregiola*		100 ^a,i^	55.89 ± 3.53 ^b,i^	14.59 ± 0.5 ^c,i^	0.63 ± 0.29 ^c,i^
ethanol	*Corregiola*		100 ^a,i^	121.59 ± 13.7 ^a,ii^	4.26 ± 2.6 ^b,ii^	0.44 ± 2.08 ^b,i^
methanol	*Corregiola*		100 ^a,i^	73.57 ± 9.33 ^b,ii^	32.83 ± 10.41 ^c,iii^	0.87 ± 0.17 ^d,i^
water	*Frantoio*		100 ^a,i^	103.19 ± 27.9 ^a,ii^	0.47 ± 0.13 ^b,ii^	0.61 ± 0.17 ^b,i^
ethanol	*Frantoio*		100 ^a,i^	122.78 ± 21.1 ^a,ii^	30.37 ± 4.48 ^b,iii^	0.87 ± 0.22 ^c,i^
methanol	*Frantoio*		100 ^a,i^	120.26 ± 9.22 ^b,ii^	41.13 ± 16.02 ^c,iii^	0.98 ± 0.56 ^c,i^
	control		100 ^a,i^			
		47.8 ± 0.1				

^a,b,c,d^ Values in the same row not having the same superscript letter are significantly different from each other. ^i,ii,iii^ Values in the same column not having the same superscript roman numeral are significantly different from each other. Values are expressed as percentage growth compared to controls with no extracts or gemcitabine. Therefore, the lower the value in response to olive leaf extract (50–200 μg/mL), the greater the anti-proliferative effect. Values greater than 100% represent cell growth greater than controls. Time = 96 h.

Interestingly, at 50 µg/mL, the water extract of the *Corregiola* variety (55.89%) had a significantly greater negative impact on the MiaPaCa-2 cells’ viability than all the other extracts (Table 3). The compounds eluting as peaks 2 and 3 in the HPLC chromatogram (Figure 1) for the *Corregiola* variety are of interest because they appear more prominent in the water extract than in the other two extracts. However, the water extract from the *Corregiola* olive tree leaves may also have other compounds which are not detected at 254 nm.

Figure 1 shows the phenolic compound profile of the olive leaf samples and it appears that oleuropein (peak 13) is by far the most abundant compound present. However, at a concentration of 200µg/mL, the crude olive leaf extracts only contained approximately 20 nM of oleuropein. Despite this very low dose, the crude leaf extracts were still able to significantly reduce the viability of the pancreatic cancer cells compared to gemcitabine at its IC_50_ (*p* < 0.05). The anti-proliferative capacity of the olive leaf extracts in the present study against pancreatic cancer cells is better than what has been observed in previous studies on cancers of different origins. Han *et al*. [22] showed that 200 µg/mL of pure oleuropein was able to dramatically reduce the cell viability of MCF-7 human breast cancer cells. Further investigation discovered that oleuropein decreased the number of MCF-7 cells by inhibiting the rate of proliferation and inducing cell apoptosis. However, the results suggest that the water extract from the *Corregiola* leaves may have other active compounds, which are more potent against this pancreatic cell line than oleuropein, since the water extracts from both olive varieties were significantly lower, not higher, in oleuropein than the 50% ethanol and 50% methanol extracts (Table 1). Therefore, the present study provides a platform for further research into olive leaf phenolic compounds and their efficacy.

## 3. Experimental Section

### 3.1. Materials

Folin Ciocalteu’s reagent, sodium carbonate, gallic acid, Sodium Nitrite, aluminium chloride, sodium hydroxide, rutin, 1,1-diphenyl-2-picrylhydrazyl (DPPH), 6-hydroxy-2,5,7,8-tetramethylchroman-2-carboxylic acid (trolox), 2,4,6-Tris(2-pyridyl)-*s*-triazine (TPTZ), ferric chloride, sodium acetate, acetic acid, copper(II) chloride, ammonium acetate (NH_4_Ac) , neocuproine methanol and ethanol were purchased from Sigma Aldrich (Castle Hill, NSW, Australia).

Human pancreatic cancer (Mia-PaCa2) cells, Dulbecco’s Modified Eagle’s Medium (DMEM), fetal bovine serum (FBS), horse serum and L-glutamine.

### 3.2. Sample Preparation and Extraction of Phenolic Compounds

**Figure 2:**
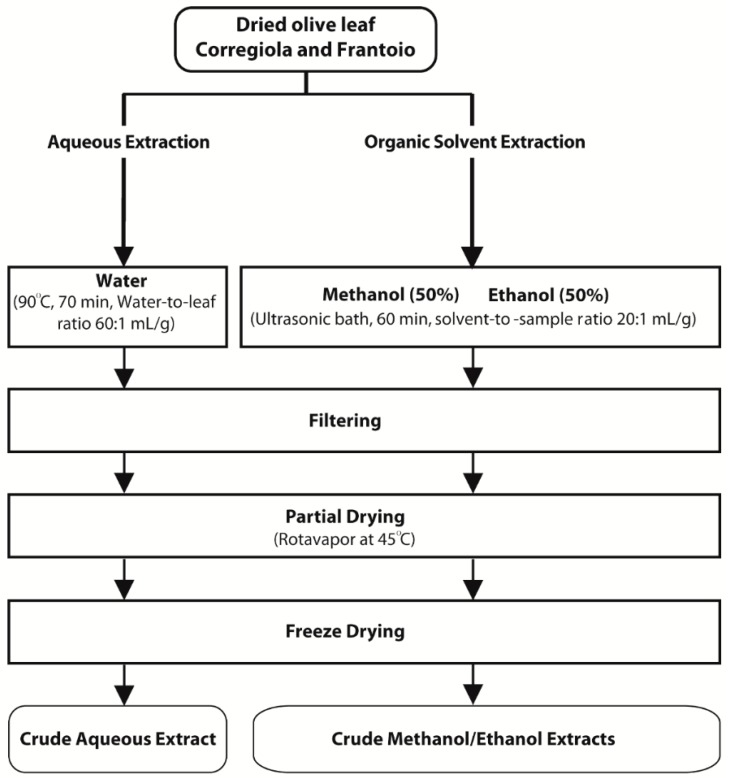
Methods for the preparation of olive leaf extracts.

*Corregiola* and *Frantoio* olive leaves were obtained from Houndsfield Estate in the Hunter Valley of NSW Australia. Leaves were dried at 120 °C for 90 min according to Ahmad-Qasem *et al*. [23], ground to a size of 0.1 mm and stored at −20 °C until further analysis. Water extracts were prepared according to Goldsmith *et al*. [24] while 50% methanol and 50% ethanol extracts were prepared according to Sahin *et al*. [19] (Figure 2). Extracts were concentrated (their volume reduced) using a rotary evaporator, freeze dried and then stored at −20 °C until further analysis.

### 3.3. Total Phenolic Compounds

The total phenolic compounds (TPC) were determined according to Thaipong *et al*. [25]. Briefly, diluted samples (300 μL) were added to Folin Ciocalteu’s reagent (300 μL) and left to equilibrate for 2 min before adding 2.4 mL of 5% sodium carbonate solution and incubated in the dark for 1 h. Absorbance was then read at 760 nm using a UV spectrophotometer (Varian, Melbourne, VIC, Australia). Gallic acid was used as the standard and results were expressed as mg of gallic acid equivalents per g of sample dry weight (mg GAE/g).

### 3.4. HPLC

The olive leaf extracts were re-dissolved at 10 mg/mL and analysed using high performance liquid chromatography (HPLC) according to Goldsmith *et al*. [26] with some minor modifications. A Shimadzu HPLC system was used (Shimadzu Australia, Rydalmere, NSW Australia) with a 250 × 4.6 mm Synergi 4 μm Fusion-RP 80A reversed-phase column (Phenomenex Australia Pty. Ltd., Lane Cove, NSW Australia) with UV detection at 254 nm. The column was maintained at 30 °C, flow rate 1 mL/min and the three solvents used for the mobile phase were: solvent A—1% acetonitrile in 0.2% H_3_PO_4_ (*v*/*v*); solvent B—100% methanol; and solvent C—100% acetonitrile. A gradient elution schedule was used. The initial solvent system at the time of injection was 96% A, 2% B and 2% C. The eluting solvent was then changed, in a linear gradient manner, to 40% A, 30% B and 30% C by 20 min and held there for 20 min. From 40–42 min, the solvent was then returned to 96% A, 2% B and 2% C and maintained there for 10 min to re-equilibrate the column with the initial solvent system before the next injection. Gallic acid was used as an internal standard.

### 3.5. Determination and Quantification of Oleuropein

The HPLC peak corresponding to oleuropein was identified using an internal standard. The quantity of oleuropein in the extracts was determined using a standard curve of oleuropein prepared in methanol, which was linear between 0.05 and 0.925 mM, with the results expressed as mmol oleuropein per g dry weight (mmol/g).

### 3.6. Flavonoids

Total flavonoids were determined according to Vuong *et al*. [27]. Briefly, powdered extracts were re-dissolved at a concentration of 1 mg/mL in their respective solvents and 0.5 mL was added to 0.15 mL of 5% sodium nitrite, incubated for 6 min before adding 0.15 mL of 10% aluminium chloride and incubating for an additional 6 min. Finally, 2 mL of sodium hydroxide was then added before incubating for a further 15 min. Absorbance was then read at 510 nm using a UV spectrophotometer (Varian, Melbourne, VIC, Australia). Rutin was used as a standard and results were expressed as mg of rutin equivalents per g of sample dry weight (mg RE/g).

### 3.7. Assessment of Antioxidant Capacity

Three assays were employed to assess the antioxidant activity of the olive leaf extracts:

#### 3.7.1. FRAP

For the ferric reducing antioxidant power (FRAP) assay, the extract was diluted and then the ferric ion reducing capacity was determined according to Thaipong *et al*. [25]. Stock solutions were: (1) 300 mM acetate buffer pH, (2) 10 mM TPTZ solution in 40 mM HCl, (3) 20 mM FeCl_3_ solution. The fresh working solution was prepared by mixing 25 mL acetate buffer, 2.5 mL TPTZ solution and 2.5 mL FeCl_3_ and then warming to 37 °C. Olive leaf extracts, trolox standards and blank (150 µL) were then added to 2.85 mL of the working FRAP solution and left to incubate in the dark at 37 °C for 30 min. Absorbance was read at 593 nm. Results were expressed as mg trolox equivalents per g of sample dry weight (mg TE/g).

#### 3.7.2. CUPRAC

For the cupric reducing antioxidant capacity (CUPRAC) assay, the extracts were diluted and their cupric ion reducing capacity was determined as described by Apak *et al*. [28]. The stock solutions were: (1) 10 mM CuCl_2_ solution, (2) ammonium acetate buffer at pH 7.0, (3) 7.5 mM neocuproine (Nc) solution in 95% ethanol. A working solution of the three reagents (1:1:1 *v*/*v*) was prepared, 3 mL of which was added to 1.1 mL of the diluted extracts, trolox standards and blanks and left to react in the dark for 1 h. Absorbance was read at 450 nm. Results were expressed as mg of trolox equivalents per g of sample dry weight (mg TE/g).

#### 3.7.3. DPPH

The free radical scavenging activity of the extracts was analyzed using the DPPH (1,1-diphenyl-2-picrylhydrazyl) assay as described by Vuong *et al*. [29]. Briefly, the appropriately diluted samples, trolox standards and blank (150 µL) were added to 2.85 mL of DPPH working solution (made to an absorbance of 1.1 ± 0.01 at 520 nm) and left to react in the dark at room temperature for 3 h. The results were expressed as % inhibition.

### 3.8. Effect of Olive Leaf Extracts on Pancreas Cells

#### 3.8.1. Pancreas Cell Culture

Human pancreatic cancer (Mia-PaCa2) cells were cultured at 37 °C under 5% CO_2_. Dulbecco’s Modified Eagle’s Medium (DMEM), supplemented with 10% fetal bovine serum (FBS), 2.5% horse serum and L-glutamine (100 µg/mL), was used.

#### 3.8.2. Assessment of Cell Growth Inhibition of Olive Leaf Extracts

Cell growth inhibition was determined using the Dojindo Cell Counting Kit-8 (CCK-8: Dojindo Molecular Technologies Inc., Rockville, MD, USA). Cells were seeded into a 96 well plate at 5 × 10^3^ cells per well and allowed to adhere for 24 h. The cells were then treated with 50–200 µg/mL of crude olive leaf extracts, positive control gemcitabine (IC_50_ = 50 nM) or vehicle control. The concentration 50–200 µg/mL was chosen in order to show the range in which the extracts had activity on the cells. This was based on previously published data on the anti-proliferative activity of olive leaf extracts for breast cancer [30]. After 96 h, 10 µL of CCK-8 solution was added before incubating at 37 °C for 120 min. The absorbance was measured at 450 nm and cell growth inhibition was determined as a percentage of control. All experiments were performed in triplicate.

### 3.9. Statistical Analysis

The one-way ANOVA and the LSD post-hoc test were used to assess mean differences in TPC levels, antioxidant capacity and cell viability between extracts using the JMP statistical software (Version 11). Data are represented as means ± standard deviations for triplicate experiments. Differences between the means were taken to be statistically significant at *p* < 0.05.

## 4. Conclusions

The development of novel extraction techniques to obtain bioactive compounds from biomass is gaining the interest of researchers as well as industry. The current study compared the phytochemical properties of six olive leaf extracts obtained from three different optimised extraction protocols: a “green” extraction method using water as a solvent, a 50% methanol extraction method and a 50% ethanol extraction protocol with the latter two also being ultrasound-assisted extraction techniques. While the TPC and antioxidant capacity of the extracts did not change depending on the extraction conditions, it is important to note that the levels of specific compounds did slightly vary and that there was a suggestion that the water extract of the *Corregiola* variety had the highest cytotoxicity of the leaf extracts against the MiaPaCa-2 pancreatic cancer cells. Although, the specific compounds causing cytotoxicity were not identified, it can be concluded that olive leaf extracts are a good source of phenolic compounds, including oleuropein. Furthermore, the olive leaf extracts at 100 and 200 μg/mL were found to significantly decrease the growth of the pancreatic cancer cells compared to the standard chemotherapeutic agent gemcitabine at its IC_50_.

This study is the first to show the anti-pancreatic cancer activity of olive leaf extracts and provides a foundation for further study of the activity of olive leaf compounds in pancreatic cancer. Moreover, this study shows the effectiveness and justifies the use of an environmentally friendly or “green” extraction method, which uses water, to extract bioactive compounds from olive leaves, including oleuropein and other phenolic compounds. This method could be easily scaled up and therefore shows great potential to benefit the olive oil production industry.

This is a preliminary study which aimed to assess the effectiveness of water as an extraction solvent for phenolic compounds from olive leaves and investigate olive leaf extracts as anti-pancreatic cancer agents. Limitations of this study include it being limited to one pancreatic cancer cell line and that the molecular mechanisms underlying activity were not investigated. Moreover, the individual compounds responsible for the anti-pancreatic cancer activity have not yet been identified. Nevertheless, this study provides a platform for further work to delineate the phenolic compound profile of olive leaf extracts as well as assess the molecular mechanisms involved in the anti-cancer activity of olive leaf extracts in pancreatic cancer cells.
